# Feasibility and usefulness of endoscopic ultrasonography-guided shear-wave measurement for assessment of autoimmune pancreatitis activity: a prospective exploratory study

**DOI:** 10.1007/s10396-019-00944-4

**Published:** 2019-04-16

**Authors:** Eizaburo Ohno, Yoshiki Hirooka, Hiroki Kawashima, Takuya Ishikawa, Hiroyuki Tanaka, Daisuke Sakai, Yoji Ishizu, Teiji Kuzuya, Masanao Nakamura, Takashi Honda

**Affiliations:** 1 Department of Gastorenterology and Hepatology, Nagoya University Graduate School of Medicine, 65 Tsuruma-cho, Showa-ku, Nagoya, 466-8550 Japan; 2 Department of Endoscopy, Nagoya University Hospital, 65 Tsuruma-cho, Showa-ku, Nagoya, 466-8550 Japan

**Keywords:** EUS elastography, Shear-wave elastography, EUS-SWM (EUS shear-wave measurement), Autoimmune pancreatitis (AIP)

## Abstract

**Purpose:**

To assess the feasibility and the clinical usefulness of a newly developed endoscopic ultrasonography (EUS) shear-wave elastography technique (EUS shear-wave measurement: EUS-SWM) in the diagnosis and treatment of autoimmune pancreatitis (AIP).

**Methods:**

Tissue elasticity was measured in the pancreas in 160 patients. The success rate of EUS-SWMs, the velocity of the shear wave (Vs, m/s), and the reliability index of the Vs measurement (VsN) were evaluated, and the elasticity (median Vs) was compared between AIP patients (*n* = 14) and normal controls.

**Results:**

A total of 3837 EUS-SWMs were performed without adverse events. Overall, 97.6% (3743/3837) were successful. The median VsN was 74%. The median Vs values of the pancreas were as follows: 2.22 m/s in the pancreatic head (push position), 2.36 m/s in the head (pull position), 1.99 m/s in the body, and 2.25 m/s in the tail. The median Vs of the AIP group (2.57 m/s) was significantly higher than that of the normal controls (1.89 m/s) (*P *= 0.0185). The mean Vs significantly decreased from 3.32 m/s to 2.46 m/s after steroid therapy (*n* = 6) (*P *= 0.0234).

**Conclusion:**

EUS-SWM is feasible and generates credible results. EUS-SWM was a useful method for assessment of the effect of steroid therapy in AIP patients.

**Electronic supplementary material:**

The online version of this article (10.1007/s10396-019-00944-4) contains supplementary material, which is available to authorized users.

## Introduction

Ultrasound elastography (US-EG) is a novel diagnostic method based on tissue characterization that enables the imaging and measurement of tissue elasticity [1, 2]. US-EG has been successfully used in various organs including the mammary gland, thyroid gland, and prostate, as well as digestive organs including the liver and pancreas [3–9]. US-EG is classified into two categories based on different mechanical properties: strain elastography (EG) and shear-wave EG. Strain EG evaluates tissue elasticity by measuring relative tissue distortion after applying pressure. Shear-wave EG is based on the properties of a shear wave and involves a Doppler-like ultrasound technique to monitor shear-wave propagation and measure the velocity of the shear wave. Theoretically, greater tissue elasticity corresponds to faster shear-wave propagation [1, 2]. By measuring shear-wave velocity (Vs), tissue elasticity can be quantified in terms of the elasticity modulus. Studies have shown that shear-wave EG can be used to noninvasively detect fibrosis in the liver and in other gastrointestinal organs via transabdominal ultrasound [10, 11]. More recently, studies have demonstrated the use of shear-wave EG to detect pancreatic fibrosis and chronic pancreatitis [12–14].

In recent years, endoscopic ultrasonography (EUS) has become an important high-resolution diagnostic technique for the detection of pancreatobiliary and gastrointestinal diseases [15]. In addition, EUS-guided fine-needle aspiration (EUS-FNA) has been used as a tool for tissue sampling. Diagnostic EUS has evolved with recent advancements in diagnostic ultrasound techniques, including contrast-enhanced EUS to evaluate blood flow and EUS elastography (EUS-EG) to evaluate tissue elasticity [16–19]. Currently, only strain EG is available with EUS; however, because strain EG provides the relative elasticity within a region of interest (ROI), it lacks objectivity in terms of reproducibility and quantification [20, 21].

Autoimmune pancreatitis (AIP) is a distinct type of pancreatitis with a hypothesized autoimmune mechanism. AIP is characterized morphologically by diffuse or focal enlargement of the pancreas and diffuse irregular narrowing of the main pancreatic duct, and serologically by increased levels of serum gamma globulin, including IgG and especially IgG4 [22]. AIP shows a marked response to steroid therapy. However, the definitions of “remission” and “relapse” are ambiguous; therefore, a method to evaluate the grade or inflammation activity of AIP is needed [23, 24].

In the present study, we performed shear-wave measurements (EUS-SWMs) using a newly developed technique for EUS-guided shear-wave EG, assessed its feasibility and capability for measuring tissue elasticity in the pancreas, and evaluated the correlation between disease activity and pancreatic elasticity in AIP patients.

## Materials and methods

### Patients

This study was approved by the Institutional Review Board of Nagoya University Hospital and was conducted in accordance with the Declaration of Helsinki. This study was registered with the University Hospital Medical Information Network (UMIN) clinical trial registry (UMIN-CTR 000028072).

A prospective study was conducted with 160 patients who underwent EUS examination and EUS-SWMs of the liver, pancreas, and other intraperitoneal organs between December 2017 and September 2018. All 160 patients were suspected of having pancreatobiliary diseases. The EUS examinations and EUS-SWM procedures were performed on the same day. All EUS-SWM procedures were performed by three experienced endosonographers (E.O, Y.H., and T.I.).

### EUS-guided shear-wave measurement (EUS-SWM) procedure

SWM is a shear-wave EG method. EUS-SWM was performed using a GF-UCT260 linear-array echoendoscope (Olympus Co., Tokyo, Japan) and an ARIETTA 850 ultrasound device (Hitachi, Ltd., Tokyo, Japan). As an elastic module, the Vs was measured for the pancreatic head [from the duodenal bulb (D1) and the descending portion of the duodenum (D2)], pancreatic body, and pancreatic tail.

**Fig. 1:**
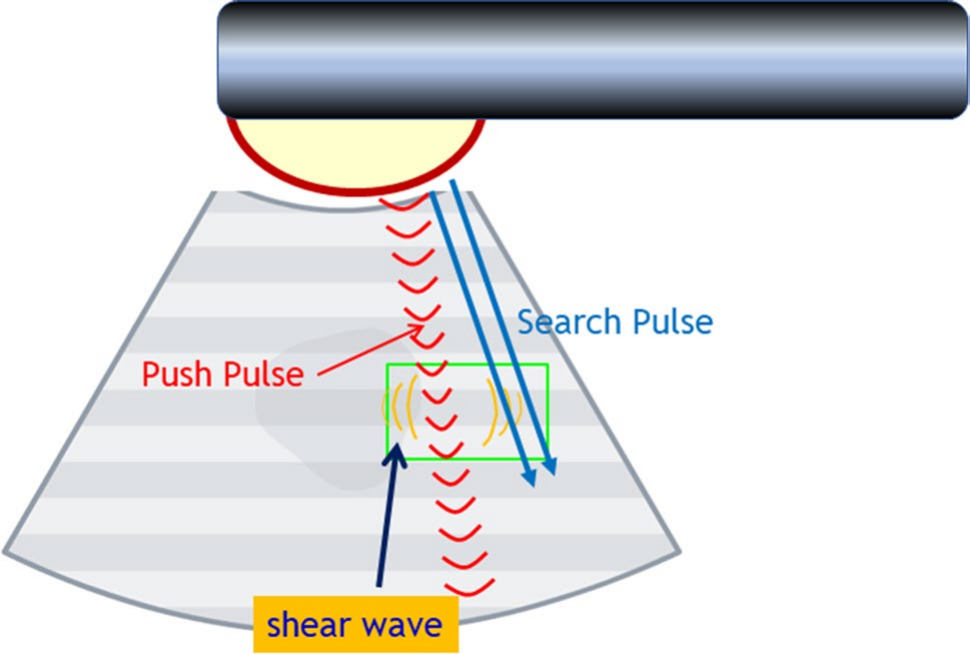
Illustration of EUS-guided shear-wave measurement. Acoustic radiation force “push pulse” is sent to the focal point in the region of interest. Shear wave is generated at the edge of push pulse and propagates off-axis. Propagation speed calculated from detection of arrival by the search pulses

**Fig. 2:**
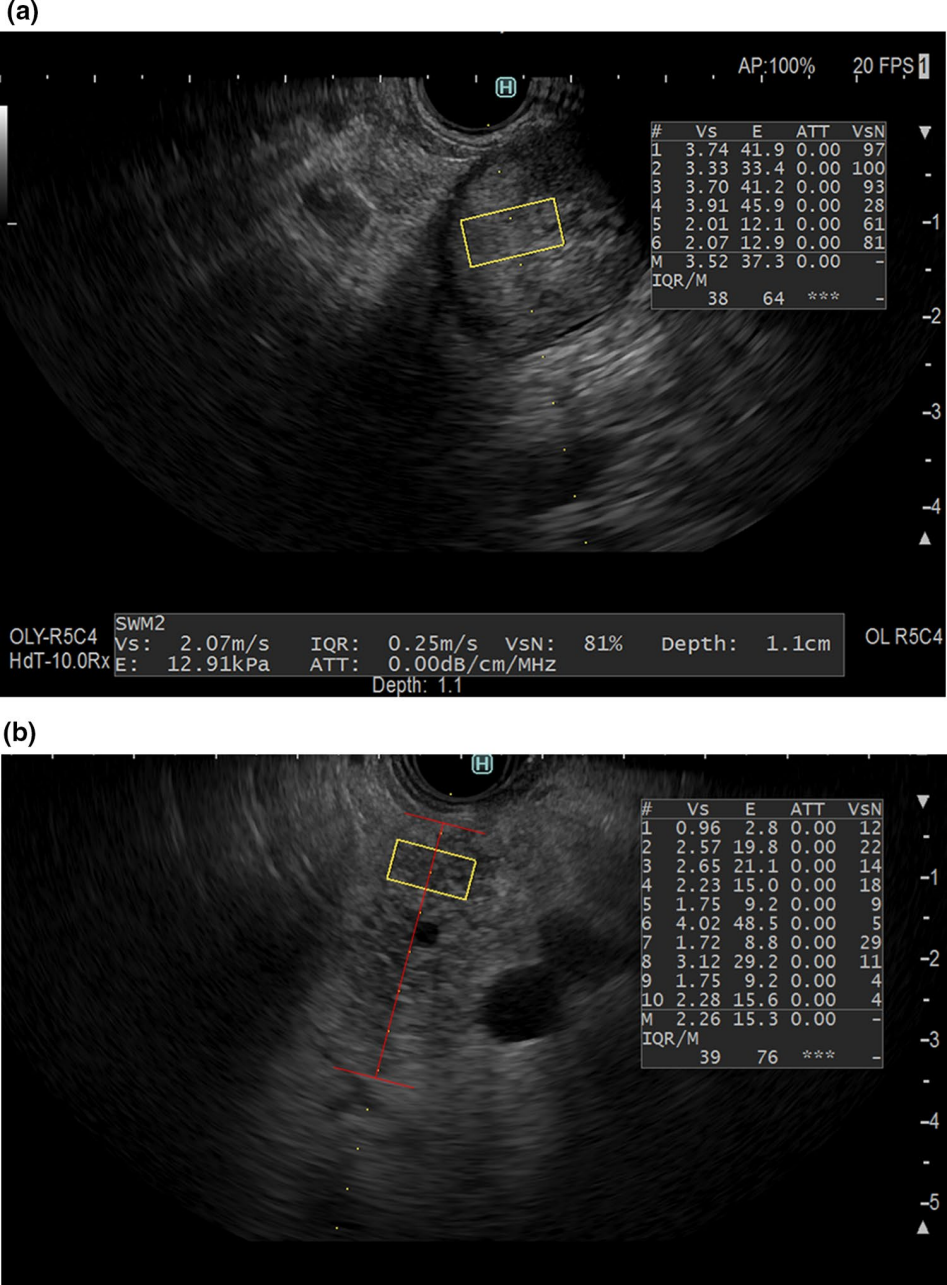
**a** A representative case of EUS-guided shear-wave measurement (EUS-SWM) in the pancreatic body of an AIP patient. The scope was positioned in the stomach. The size of the region of interest (ROI) was 5 × 10 mm. The measured data are displayed at the bottom of the screen. The measured shear-wave velocity (Vs) was 2.07 m/s, and the reliability index (VsN) was 81%. **b** A representative case of measurement of size of pancreatic parenchyma. We measured the size of the pancreas (length of red line) at the measurement site of EUS-SWM

If a patient had any solid pancreatic lesions, then we also performed EUS-SWM of the solid mass and pancreatic parenchyma out of the lesion. The Vs was displayed in meters per second or kilopascals (kPa) through Young’s modulus *E *= 3*(*Vs^2^*ρ)*, where *E* is Young’s modulus, Vs is the shear-wave velocity, and *ρ* is the tissue density. Stiffer tissue corresponds to faster shear-wave propagation (Fig. 1). Using the reliability index, the percentage of the net amount of effective shear-wave velocity (VsN: %) was calculated to determine whether shear-wave propagation was detected correctly and whether unnecessary components other than those generated by shear-wave propagation existed in the ROI according to predefined rejection conditions, and the value was displayed on the monitor [10, 11]. EUS-SWMs of the pancreatic body and tail were performed through the stomach, and measurements of the pancreatic head were performed through the duodenum. Measurements were performed during minimal respiratory fluctuation to avoid breathing artifacts as much as possible. A rectangular 5 × 10-mm ROI (height × width) was used in most cases and was set at 5–10 mm below the EUS probe. ROIs were set to exclude vessels, the bile duct, the pancreatic duct, and cystic lesions. In each pancreatic region, EUS-SWM was measured up to 10 times and at least five times (until VsN ≥ 50% was obtained five times). We also measured the size of pancreatic parenchyma at the measurement site of the EUS-SWM (Fig. 2a, b). The success rate of EUS-SWM was defined as the percentage of the measured value of Vs displayed on the monitor. The success rates of EUS-SWM, Vs, and VsN were evaluated.

### Comparison of pancreatic elasticity of AIP patients and normal controls

To elucidate the use of EUS-SWM for diffuse pancreatic lesions, the median elasticity (median Vs) of the pancreatic body was compared between AIP patients and normal controls. A normal control was defined as a patient without abnormal EUS findings in the pancreas according to the Rosemont criteria. For the comparison of pancreatic elasticity between normal controls and AIP patients, data were obtained from the pancreatic body through the stomach, because EUS-FNA was performed at the pancreatic body, which was expected to exhibit minimal endoscopic compression effects on the pancreas and reduce respiratory artifacts.

Additionally, for AIP patients who received steroid therapy (*n* = 6), the pancreatic elasticity, the size of the pancreatic body on EUS and CT, and a serological marker (serum IgG4) were compared before and 2 weeks after the administration of corticosteroids.

### Statistical analysis

Descriptive statistics are expressed as the medians and IQR. Qualitative variables were compared by *χ*^2^ tests, and quantitative variables were compared using the Mann–Whitney *U* test. Comparisons of pancreatic elasticity and serum IgG and IgG4 levels before and after steroid therapy were evaluated by the Wilcoxon signed-rank test. Statistical analysis was performed using JMP9 for Windows (SAS Institute Inc., Cary, North Carolina, USA). All tests were 2-tailed, and *P *< 0.05 was considered significant.

## Results

Table 1 summarizes the characteristics of the 160 patients who underwent the EUS-SWM procedure [95 males, median age: 64.7 (IQR 57–75)]. One hundred and forty-four patients had the following pancreatic diseases: focal pancreatic lesions including intraductal papillary mucinous neoplasm (IPMN) (*n* = 70), pancreatic cancer (PC) (*n* = 18), serous cystic neoplasm (SN) (*n* = 9), pancreatic neuroendocrine neoplasm (PanNEN) (*n* = 5), solid pseudopapillary neoplasm (SPN) (*n* = 3), ampullary tumor (*n* = 3), and metastatic pancreatic tumor (*n* = 1); and diffuse pancreatic lesions including autoimmune pancreatitis (*n* = 14: six patients were examined before and after the steroid therapy), chronic pancreatitis (*n* = 17), and early stage of chronic pancreatitis (*n* = 4). Sixteen patients had a normal pancreas. None of the patients who underwent EUS-SWM experienced any adverse events.

**Table 1:** Patient characteristics

Gender (male/female)	95/65
Age (median IQR), y	68 (57–75)

Diagnosis	(*n*)
Normal pancreas	16
Diffuse pancreatic lesion	
AIP	14
CP	17
eCP	4
Focal pancreatic lesion	
IPMN	70
PC	18
SN	9
PanNEN	5
SPN	3
Ampullary tumor	3
Metastasis	1

Normal pancreas defined as patients without abnormal EUS findings in the pancreas

*IQR* interquartile range, *AIP* autoimmune pancreatitis, *CP* chronic pancreatitis, *eCP* early stage of chronic pancreatitis, *IPMN* intraductal papillary mucinous neoplasm, *PC* pancreatic cancer, *SN* serous neoplasm, *PanNEN* pancreatic neuroendocrine neoplasm, *SPN* solid pseudopapillary neoplasm

### Feasibility of EUS-SWM for the pancreas

A total of 3837 EUS-SWMs were obtained, with 97.6% (3743/3837) of the measurements performed successfully. No significant difference in the success rate of EUS-SWM was observed among different locations, with rates of 98.1, 96.9, 96.3, and 98.8% in the head (D1), head (D2), body, and tail of the pancreas, respectively (*P *= 0.4997, Table 2). Measurement failures were cases in which measured values were not displayed for certain measurements. Artifacts due to respiratory movements at the site of measurement, including surrounding ductal structures such as pancreatic ducts or vessels in the ROI, and the tissue heterogeneity in the measurement area were considered to be the causes of measurement failure.

**Table 2:** Results of shear-wave velocity (Vs) measurement

Location	Number of cases	Number of measurements	Median Vs (IQR) m/s	Median VsN (IQR) %	Rate of cases with VsN > 50%	Success rate (%)
Elasticity of the pancreas						
Ph D1	124	883	2.22 (1.77–2.78)	83 (48–100)	73.9	98.1% (866/883)
Ph D2	100	712	2.36 (1.70–2.85)	75 (41–98)	70.1	96.9% (690/712)
Pb	155	1130	1.99 (1.51–2.53)	74 (40–96)	67.5	96.3% (1088/1130)
Pt	132	1112	2.22 (1.77–2.78)	83 (48–100)	61.2	98.8% (1099/1112)
Total	160	3837	2.21 (1.65–2.82)	74 (38–97)	67.6	97.6% (3743/3837)

*D1* measured from the duodenal bulb, *D2* measured from the descending part of the duodenum

**Fig. 3:**
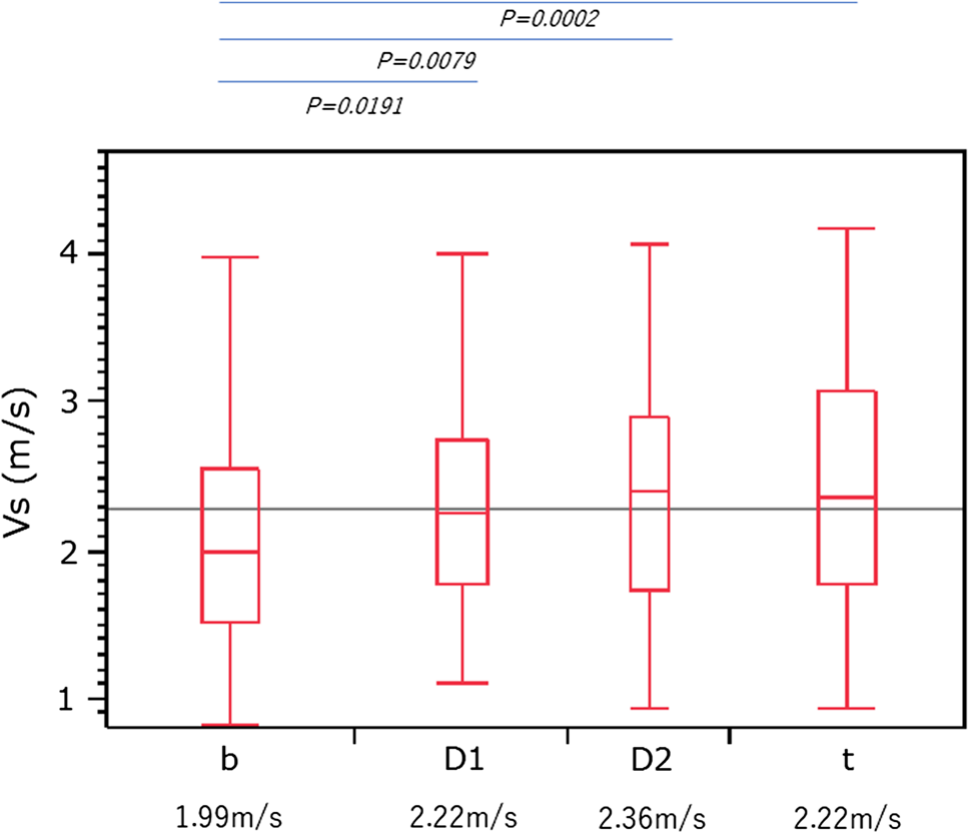
The measured shear-wave velocity (Vs) in the pancreas. The elasticity of the pancreatic body was significantly lower than that in other pancreatic areas, *b* pancreatic body, *D1* pancreatic head from the duodenal bulb, *D2* pancreatic head from the descending portion of the duodenum, *t* pancreatic tail

The median Vs values for all measurements of each of the locations were as follows: 2.22 m/s (IQR 1.77–2.78) in the head (D1), 2.36 m/s (IQR 1.70–2.85) in the head (D2), 1.99 m/s (IQR 1.51–2.53) in the body, and 2.22 m/s (IQR 1.77–2.78) in the tail of the pancreas. The elasticity of the pancreatic body was significantly lower than the elasticity in other pancreatic areas (Fig. 3).

**Fig. 4:**
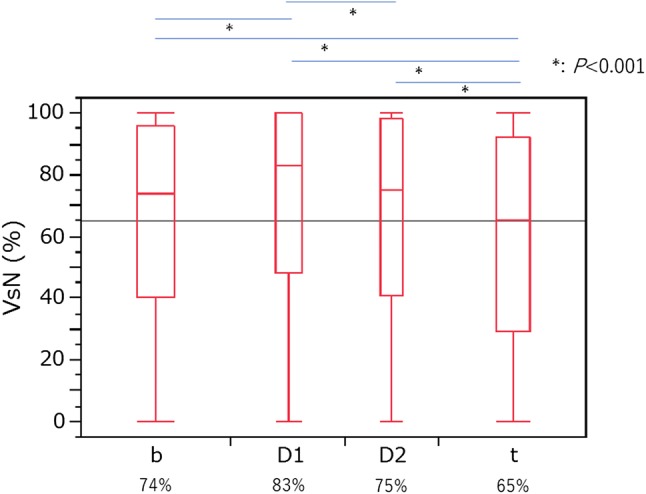
The measured reliability index (VsN) in the pancreas. Relationship between VsN and the location of measurement. VsN tended to be higher at the pancreatic head and body

The median VsN values for each location were as follows: 83% (IQR 48–100) in the head (D1), 75% (IQR 41–98) in the head (D2), 74% (IQR 40–96) in the body, and 65% (IQR 29–92) in the tail of the pancreas (Fig. 4).

The median reliability index of EUS-SWM (VsN) was 63% (IQR 59), and this value was 67.6% (2595/3837) for the measurements with a relatively high VsN score (≥ 50%) (Table 2). The median sizes (IQRs) of pancreatic parenchyma measured by EUS in all subjects were 18 mm (15.3–20) for the head (D1),18 mm (13–20) for the head (D2), 16 mm (12–19) for the body, and 13 mm (11–15) for the tail of the pancreas. The relationship between the pancreatic elasticity (Vs) and the size of pancreatic parenchyma at the measurement site is shown in Supplementary Fig. 1. There was no clear correlation between pancreatic size and pancreatic elasticity.

### Comparison of pancreatic elasticity of AIP patients and normal controls

To elucidate the utility of EUS-guided SWM, we performed measurements and comparative analysis of the pancreatic elasticity in patients with diffuse pancreatic lesions that were expected to be histologically homogeneous.

**Fig. 5:**
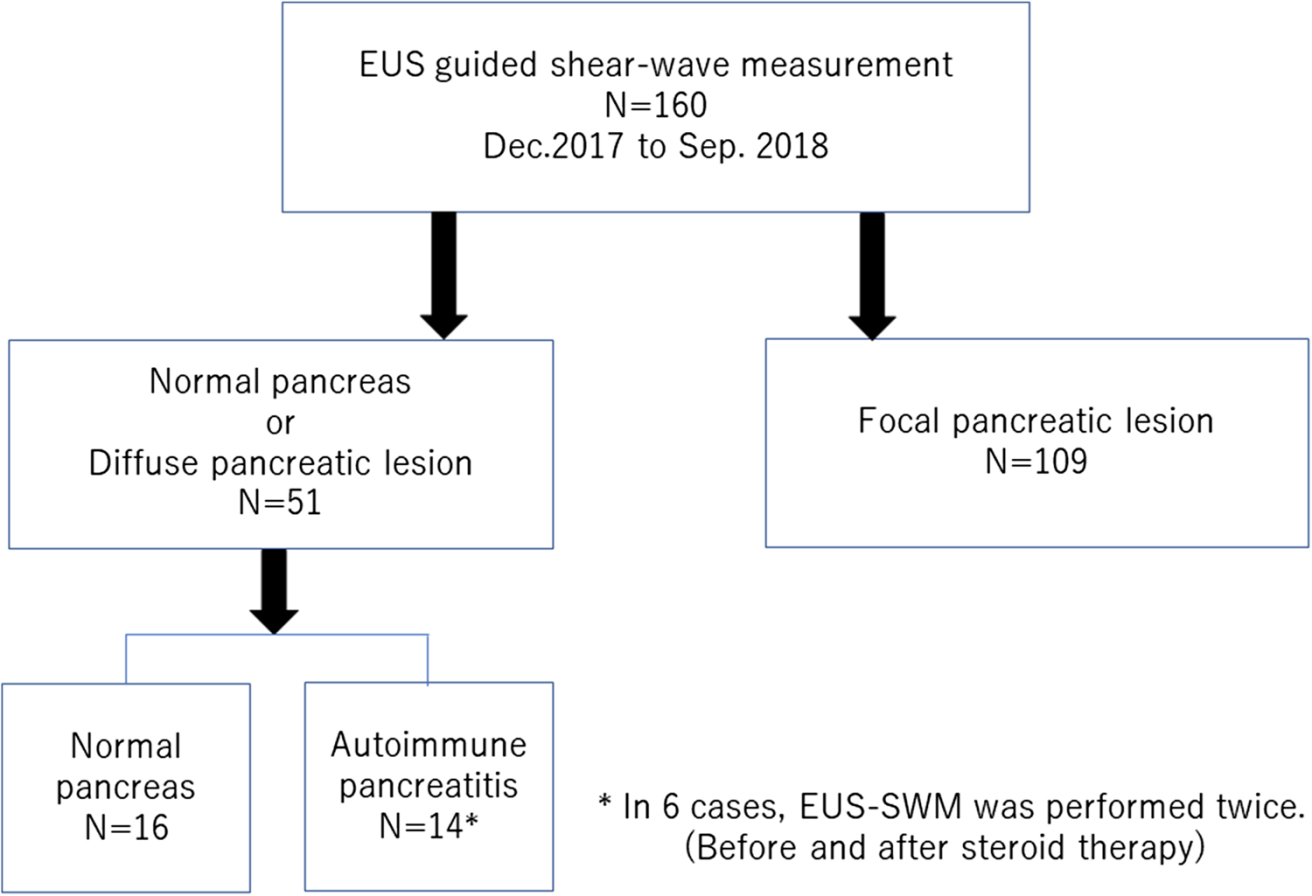
Patient flow diagram

**Fig. 6:**
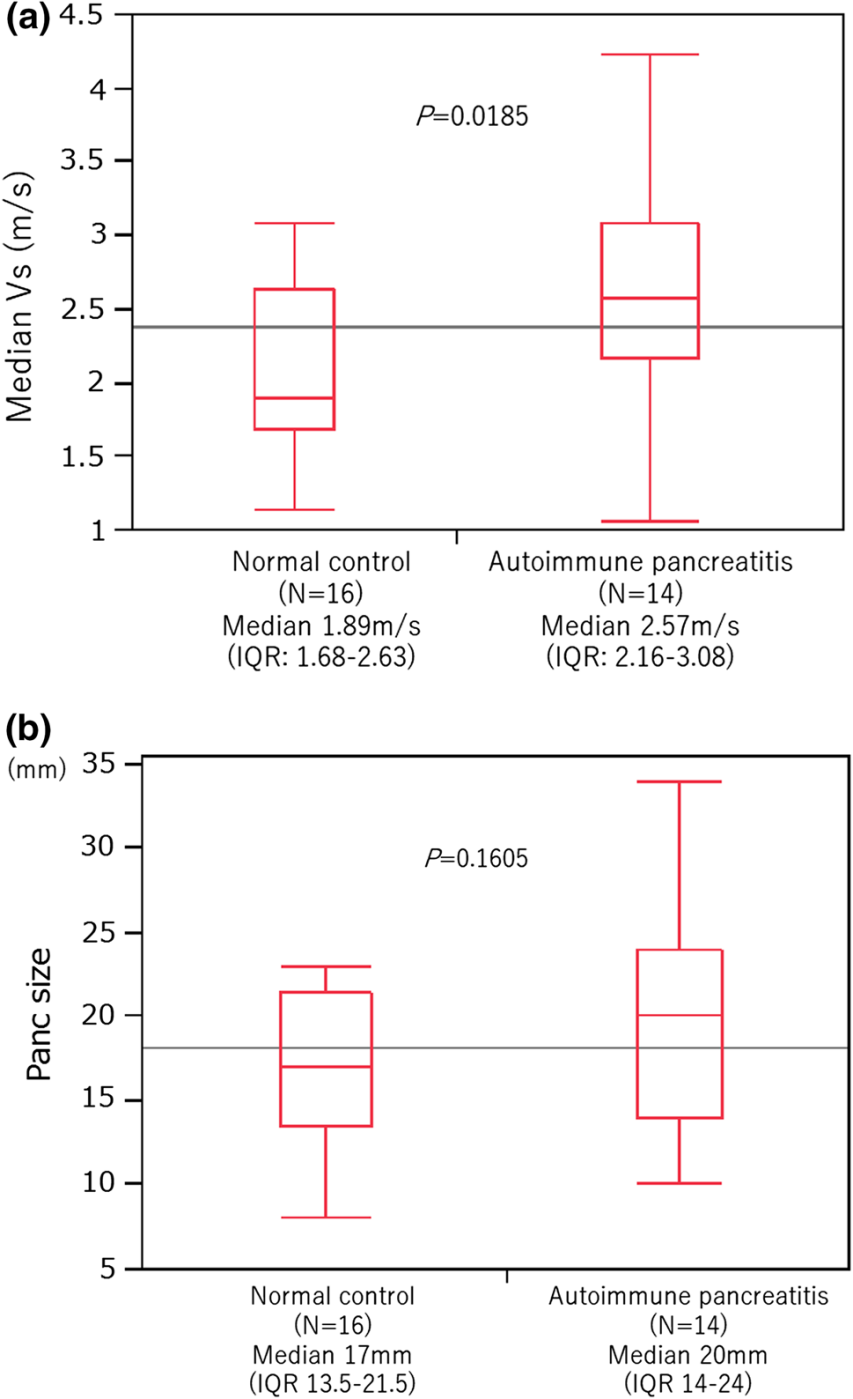
**a** The median Vs of the pancreatic body in patients with autoimmune pancreatitis (AIP) and in normal controls. The medial elasticity of AIP patients was significantly higher than that of normal controls. **b** The median size of the pancreatic body in patients with AIP and in normal controls. There was no significant difference between AIP and normal control

**Fig. 7:**
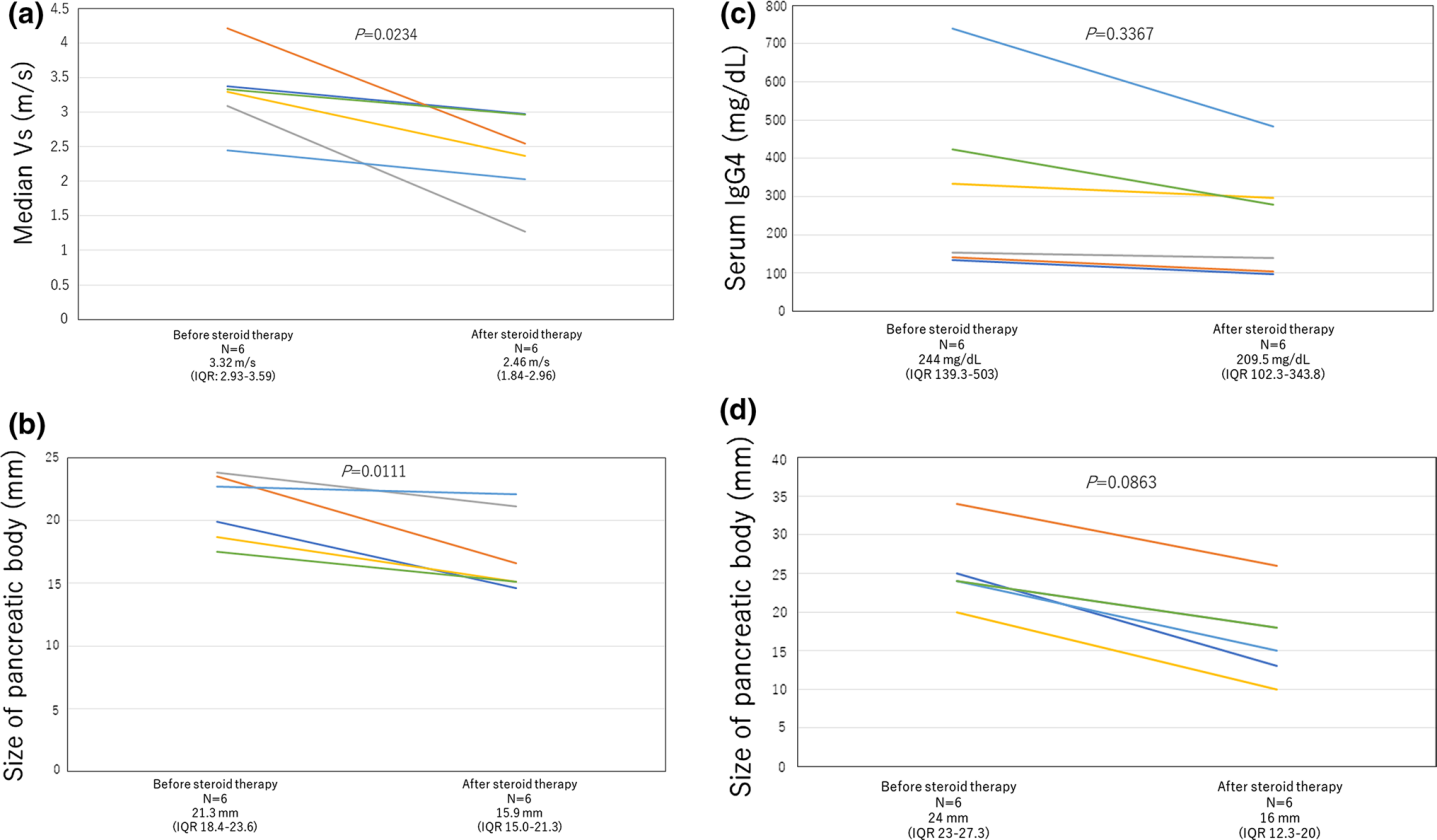
**a** Changes in the elasticity of the pancreas (Vs) before and after steroid treatment. The median Vs decreased significantly after administration of corticosteroids. **b** Changes in the size of the pancreatic body on CT before and after steroid treatment. The size of the pancreatic body on CT decreased significantly after steroid therapy. **c** Changes in serum IgG4 level before and after steroid treatment. Serum IgG4 levels did not decrease in the 2 weeks after administration of corticosteroids. **d** Changes in the size of the pancreatic body measured by EUS before and after steroid treatment. The size of the pancreatic body showed a decreasing trend, but there was no statistically significant difference after steroid therapy

Eight AIP patients (in six cases, EUS-SWM was performed twice) and 16 normal controls were included in this analysis (Fig. 5). All AIP patients had definitive type 1 AIP according to the International Consensus Diagnostic Criteria (ICDC) [22]. The median ages in the AIP group and normal control group were 71 and 63.5 years, respectively (*P *= 0.3276) (Table 3). The median Vs in the pancreatic body of the AIP group was 2.57 m/s (IQR 2.16–3.08), which was significantly higher than that of the normal control group [1.89 m/s (IQR 1.68–2.63)] (*P *= 0.0185) (Fig. 6a, b). For the patients who received steroid therapy (*n* = 6), EUS-SWM was performed before and 2 weeks after corticosteroid administration (prednisone 0.6 mg/kg/day). The remaining two patients were cases of relapse of AIP who had already received steroids. The mean Vs decreased significantly from 3.32 m/s (IQR 2.93–3.59) before to 2.46 m/s (1.84–2.96) after steroid therapy. The size of the pancreatic body on CT decreased significantly (21.3 mm vs 15.9 mm). The serum IgG4 level (244 mg/dl vs 209.5 mg/dl, *P *= 0.3367) and the size of the pancreatic body on EUS (24 mm vs 16 mm, *P *= 0.0863) showed a decreasing trend, but no statistically significant difference was observed 2 weekjs after the administration of steroid therapy (Fig. 7a–d). There was no significant correlation between Vs and the serum IgG4 level in AIP patients (Supplementary Fig. 2).

**Table 3:** Characteristics of patients with autoimmune pancreatitis

Gender (male/female)	7:1
Age (median IQR), y	71 (57–76)
Naïve:Relapse	6:2
Symptom	Jaundice/Cholangitis 5
	Abdominal discomfort 1

## Discussion

Since EUS-EG was first described by Giovannini in 2006, several studies have demonstrated its use in pancreatic diseases [19]. Currently, only strain EG has been applied in EUS [25–27]. Since strain EG measures the relative elasticity within an ROI, it does not provide an absolute measurement of tissue elasticity. Therefore, EUS-EG results have been examined qualitatively by pattern recognition methods and quantitatively by measuring the strain ratio and performing strain histogram analysis. In recent years, the use of newly developed analysis programs such as neural network analysis and fractal geometry analysis has been reported [28–34]. However, shear-wave EG by transabdominal ultrasound has become a standard method for the assessment of liver fibrosis, and application of shear-wave EG to EUS is anticipated. An advantage of EUS-SWM is that it is possible to measure the objective elastic value immediately and repeatedly.

In this study, we evaluated the feasibility of EUS-SWM for the liver and pancreas, which can be measured with the ARIETTA 850 (Hitachi) device. Currently, the only ultrasonic endoscopic probe that can be used for EUS-SWM is the UCT 260 linear probe (Olympus). The ARIETTA 850 not only enables SWM via transabdominal ultrasound scan but also enables measurement of the VsN to evaluate the reliability of the measured Vs. The SWM device has a unique reliability index, the VsN, which is used to determine whether the Vs value is reliable. Yada reported that a VsN ≥ 50% could be a good indicator of accurate SWM in the liver [10, 11]. Using this index, data can be collected with high reliability.

To elucidate the utility of EUS-guided SWM, we measured and performed a comparative analysis of the pancreatic elasticity in patients with diffuse pancreatic lesions that were expected to be histologically homogeneous. Our results indicate that pancreatic elasticity was significantly decreased after administration of steroids.

The present study is the first report to show that pancreatic elasticity could be a more sensitive measure of disease activity than serum markers during the course of treatment of AIP patients. Additionally, EUS-SWM can provide the absolute elastic modulus, which can be used to directly compare the elasticity between individuals. EUS-SWM may be a promising and useful EG method for measurement of the elasticity of the pancreas. Corticosteroids have been established as the standard therapy for AIP. Remission of AIP or treatment response is usually evaluated by improvement of pancreatic swelling based on imaging findings such as CT. Although it has been reported that the serum IgG4 level after introduction of steroid therapy is useful as a predictor of AIP relapse, it has not been established as an early marker for determination of therapeutic effect [35]. Tabata et al. reported that serum IgG4 levels normalized in 17% of AIP patients at 1 month, 46% at 3 months, and 46% at 12 months after starting steroid therapy [36]. In the present study, we evaluated the changes in serum IgG4 levels and pancreatic size and elasticity at the early phase of 2 weeks after the introduction of steroid therapy according to the steroid trial’s early evaluation criteria in the ICDC guidelines [22].

The present study was a preliminary trial aiming to assess the feasibility of EUS-SWM. Therefore, there were some limitations. First, the primary endpoint of this study was the feasibility of EUS-SWM for the pancreas. For the majority of the patients who underwent EUS, the aim was to examine the pancreatic lesion (e.g., pancreatic cyst), and the number of normal pancreas cases was small. In addition, the number of patients with diffuse pancreatic disease was also limited. Furthermore, causes of pancreatic diseases may strongly affect the hardness of the pancreatic parenchyma, such as AIP and chronic pancreatitis; therefore, we included the measurement results for patients with focal pancreatic lesions except for focal mass lesions.

Second, when performing EUS-SWM, fluctuations due to respiration and the compression effect on the target organ by the EUS scope position must be considered.　 In transabdominal ultrasound SWM, wave refraction or dispersion may be induced by the histological heterogeneity of the lesion, or a capsule-like structure may cause inaccurate assessment or failure of the elasticity measurement.

In the present study, the EUS-SWM system yielded a low reliability index (VsN) or measurement failure for extremely hard lesions with a heterogeneous structure such as fibrosis in a tumor. Various artifacts have been reported to appear due the position of the measurement ROI, effects of precompression by the probe, motion effect of the target lesion, and the heterogeneity in the focal mass of the lesion, with regard to hardness quantification via SW-EG [37–42].

Third, we did not compare tissue elasticity determined by EUS-SWM with the corresponding pathological findings. Since we performed tissue diagnosis by EUS-FNA only before the introduction of steroids, we could not evaluate the correlation of histological findings with pancreatic elasticity. To consider the rapid response for decreasing pancreatic elasticity, we presumed that the degree of infiltration of inflammatory cells into the pancreatic parenchyma affected the pancreatic elasticity in AIP patients. Future studies should compare the measured tissue elasticity levels with pathological findings, such as the degree of pancreatic fibrosis and tumor cell density, to determine the clinical utility of the tissue elasticity measurement.

Among the patients in the present study, the Vs could not be measured in one patient with a pancreatic lesion associated with acute lymphocytic leukemia. Since the pancreatic parenchyma of this patient was extremely hypoechoic, this finding suggests that the sensitivity of EUS-SWM may be reduced in regions with extremely low echogenicity. Similarly, although SWM was performed by transabdominal ultrasound, a shear wave could not be measured in this patient. Barr reported that the shear wave was detected by the ultrasonic echo signal. Therefore, when areas in B-mode images show an extremely low signal, they indicate that the echo signal is too low for successful detection [39]. With the present EUS-SWM system, the upper limit of measurable Vs was set as 5.0 m/s. We hypothesized that this system may be limited in the case of SWMs in extremely low-echogenic regions or high-elasticity regions [37–40].

Our findings suggest that EUS-SWM may be challenging in some lesions or tissues, and measurements may fluctuate due to breathing and pressure artifacts. Therefore, future studies should focus on establishing optimal scanning methods, imaging settings, and image analysis methods.

## Conclusion

In conclusion, this is the first report of EUS-SWM for the pancreas. EUS-SWM was feasible and useful for assessment of the effect of steroid therapy in AIP patients. EUS-SWM is a promising method that may facilitate the implementation of EUS-EG in the future.

## Electronic supplementary material

Below is the link to the electronic supplementary material.

Supplementary material 1 (TIFF 89 kb)

Supplementary material 2 (TIFF 92 kb)

Supplementary material 3 (TIFF 87 kb)

Supplementary material 4 (TIFF 86 kb)

Supplementary material 5 (TIFF 96 kb)
