# Supplementary figures and images for: Feasibility and usefulness of endoscopic ultrasonography-guided shear-wave measurement for assessment of autoimmune pancreatitis activity: a prospective exploratory study
Source: J Med Ultrason (2001). 2019 Apr 16;46(4):425–33. doi: 10.1007/s10396-019-00944-4 (PMC6765472; doi:10.1007/s10396-019-00944-4)

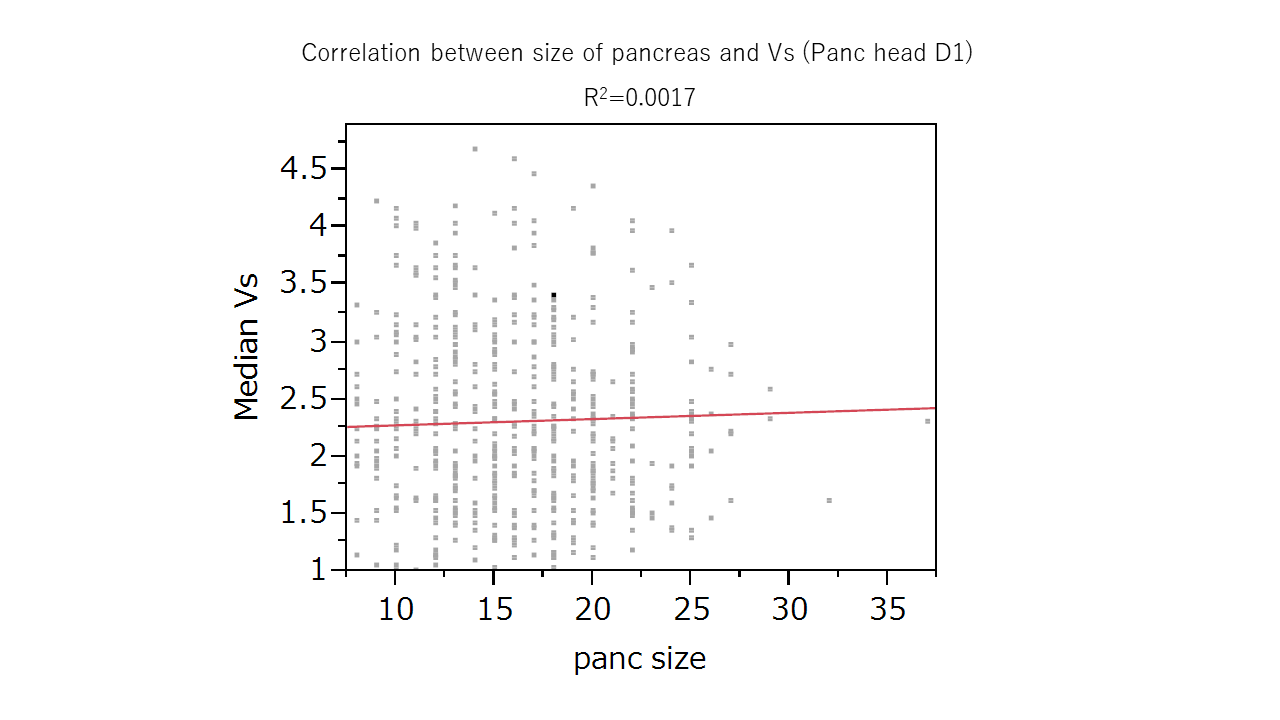

Supplement: Supplementary file 1 — Supplementary material 1 (TIFF 89 kb) [file 10396_2019_944_MOESM1_ESM.tif]

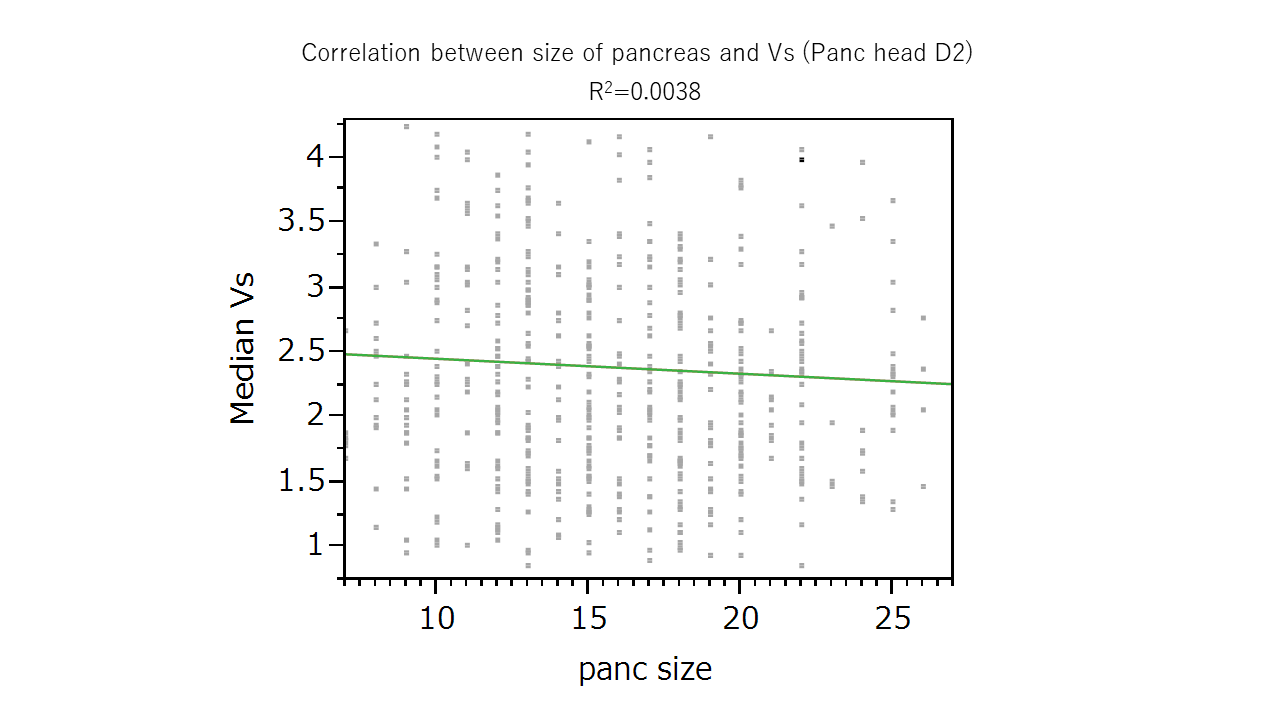

Supplement: Supplementary file 2 — Supplementary material 2 (TIFF 92 kb) [file 10396_2019_944_MOESM2_ESM.tif]

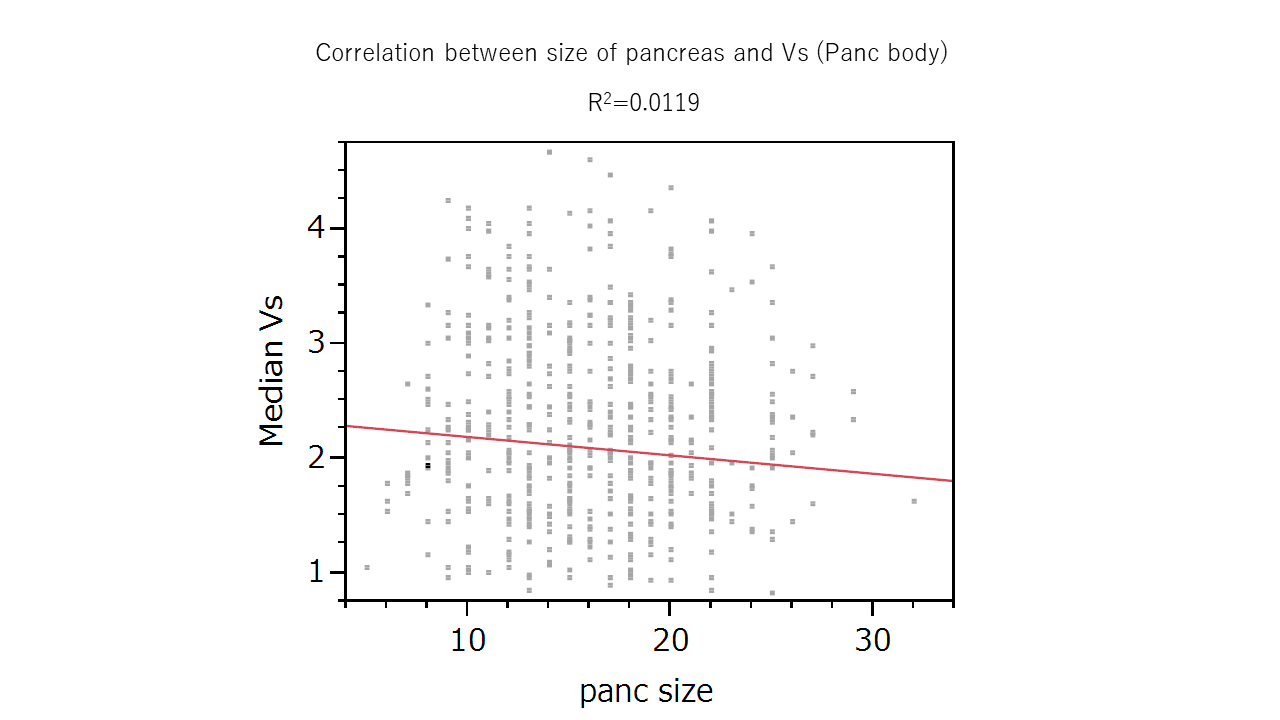

Supplement: Supplementary file 3 — Supplementary material 3 (TIFF 87 kb) [file 10396_2019_944_MOESM3_ESM.tif]

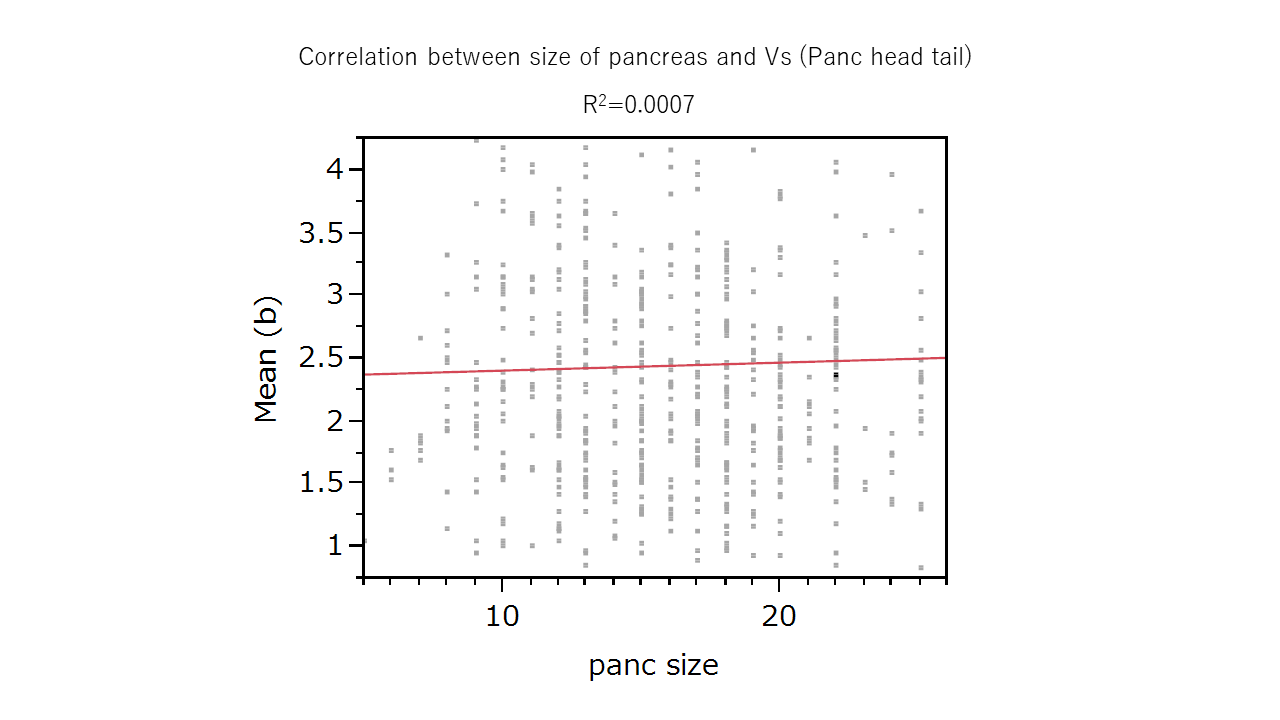

Supplement: Supplementary file 4 — Supplementary material 4 (TIFF 86 kb) [file 10396_2019_944_MOESM4_ESM.tif]

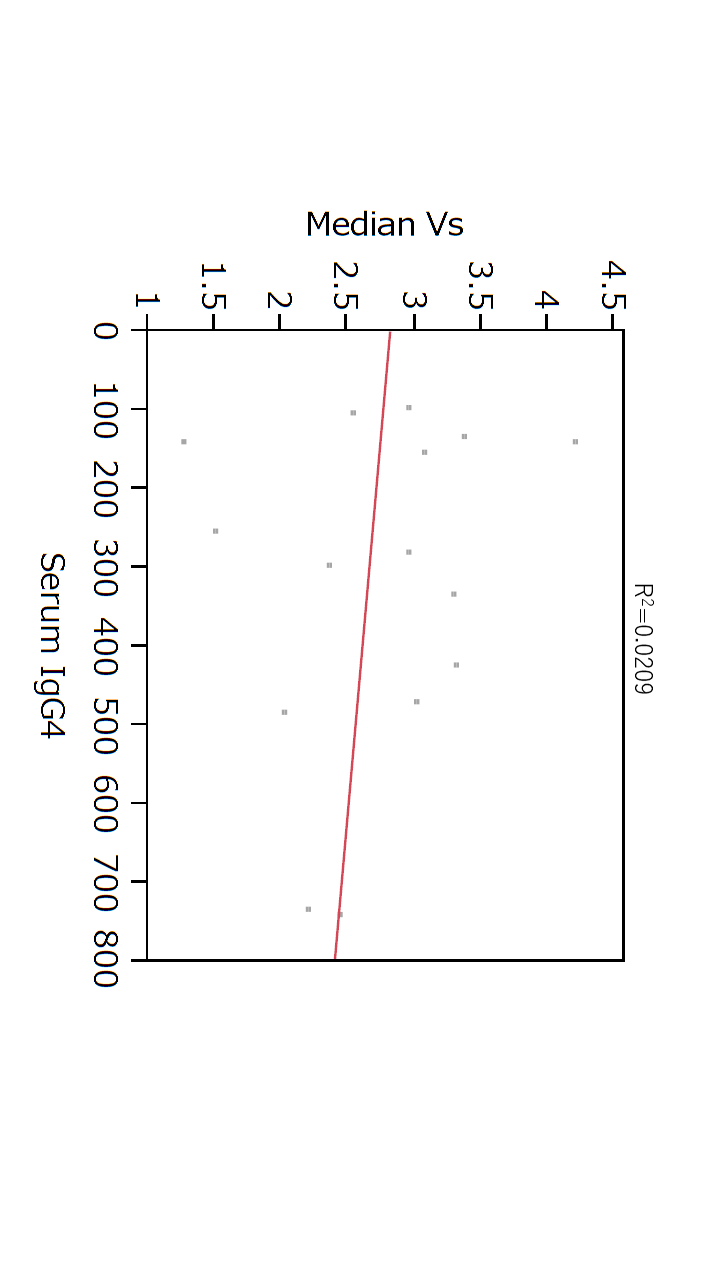

Supplement: Supplementary file 5 — Supplementary material 5 (TIFF 96 kb) [file 10396_2019_944_MOESM5_ESM.tif]
